# Diabetes Mellitus, Exercício Físico e Variabilidade da Frequência Cardíaca

**DOI:** 10.36660/abc.20220902

**Published:** 2023-01-31

**Authors:** Gustavo Augusto Ferreira Mota, Mariana Gatto, Luana Urbano Pagan, Suzana Erico Tanni, Marina Politi Okoshi

**Affiliations:** 1 Departamento de Clínica Médica Faculdade de Medicina de Botucatu Universidade Estadual Paulista Botucatu SP Brasil Departamento de Clínica Médica – Faculdade de Medicina de Botucatu – Universidade Estadual Paulista (UNESP), Botucatu, SP – Brasil

**Keywords:** Diabetes Mellitus/prevenção e controle, Exercício, Frequência Cardíaca, Fatores de Risco, Obesidade, Comportamento Sedentário, Idoso

A prevalência de diabetes mellitus (DM) tem crescido nas últimas décadas.^1^ Fatores de risco para desenvolver DM foram bem estabelecidos em estudos clínicos. Entre esses, destacam-se o sedentarismo, envelhecimento e obesidade.^2^ O DM, definido pelo aumento da concentração plasmática de glicose, é comumente acompanhado por alterações cardiovasculares como hipertensão, doença coronariana, insuficiência cardíaca e neuropatia autonômica cardíaca.^3^ Como as doenças cardiovasculares constituem as principais causas de óbito e morbidade na atualidade, estudos que visem o melhor entendimento dos mecanismos fisiopatológicos inerentes ao DM têm grande importância.

A neuropatia autonômica cardíaca é caracterizada por alterações em fibras que inervam o coração e vasos sanguíneos.^4 , 5^ Nesta condição, alterações da função nervosa levam a redução da variabilidade da frequência cardíaca e alteração da sensibilidade barorreflexa.^6^ O exercício físico é considerado uma terapia não farmacológica importante para auxiliar na prevenção e controle do DM e de suas consequências clínicas.^7^ Nos últimos anos, pesquisadores vêm observando que o exercício pode ser eficaz também na prevenção e tratamento da neuropatia autonômica cardíaca.^7^

O exercício físico aumenta a variabilidade da frequência cardíaca e melhora a função autonômica em condições fisiológicas e patológicas.^8^ Estudos experimentais em DM mostraram que o exercício diminui a bradicardia e melhora a função do sistema nervoso parassimpático.^9 , 10^ No entanto, os efeitos do exercício sobre a variabilidade da frequência cardíaca no DM não foram completamente esclarecidos. Souza Neto et al.^11^ investigaram, em ratos diabéticos, os efeitos do exercício físico na variabilidade da frequência cardíaca, capacidade funcional, e variáveis funcionais cardíacas obtidas em preparações de coração isolado. Os autores utilizaram o modelo experimental de DM tipo 1 induzido por estreptozotocina. Os animais foram submetidos a dois tipos de exercício, o treinamento intervalado de alta intensidade (HIIT), alternando ciclos de 1:1 min entre 50% e 90% da capacidade máxima de exercício, e o treinamento contínuo, realizado a 70% da capacidade máxima de exercício. Ratos sem DM e com DM sem treinamento constituíram os grupos controle. Eletrocardiogramas foram registrados por uma hora sem restrição dos animais. Após 4 semanas, o HIIT induziu maior melhora da capacidade funcional que o treinamento contínuo, tanto nos ratos controle como naqueles diabéticos. A função cardíaca foi reduzida nos grupos com DM e não foi influenciada pelo exercício físico. Os grupos com DM tiveram menor frequência cardíaca que os controles, independentemente da realização e do tipo de exercício. Apesar da menor frequência cardíaca nos ratos diabéticos, a variabilidade da frequência cardíaca não diferiu entre os grupos DM e controles.

Como os autores enfatizaram, a curta duração do estudo^11^ pode ter sido uma limitação, uma vez que efeitos decorrentes do DM e do exercício físico sobre a variabilidade da frequência cardíaca poderiam ter ocorrido se houvesse tempo maior de observação.

Os resultados controversos ao da literatura mostram a necessidade de estudos adicionais para esclarecer os efeitos do treinamento físico de alta intensidade e do treinamento contínuo na variabilidade da frequência cardíaca durante o DM.

## References

[1] Tsao CW, Aday AW, Almarzooq ZI, Alonso A, Beaton AZ, Bittencourt MS (2022). Heart Disease and Stroke Statistics-2022 Update: A Report From the American Heart Association. Circulation.

[2] Gimenes R, Gimenes C, Rosa CM, Xavier NP, Campos DHS, Fernandes AAH (2018). Influence of apocynin on cardiac remodeling in rats with streptozotocin-induced diabetes mellitus. Cardiovasc Diabetol.

[3] Ahmad E, Lim S, Lamptey R, Webb DR, Davies MJ (2022). Type 2 diabetes. Lancet (London, England)..

[4] Forbes JM, Cooper ME (2013). Mechanisms of diabetic complications. Physiol Rev.

[5] Vinik AI, Erbas T, Casellini CM (2013). Diabetic cardiac autonomic neuropathy, inflammation and cardiovascular disease. J Diabetes Investig.

[6] Souza SBC, Flues K, Paulini J, Mostarda C, Rodrigues B, Souza LE (2007). Role of exercise training in cardiovascular autonomic dysfunction and mortality in diabetic ovariectomized rats. Hypertens.

[7] Singleton JR, Smith AG, Marcus RL (2015). Exercise as Therapy for Diabetic and Prediabetic Neuropathy. Curr Diab Rep.

[8] Grisé KN, Olver TD, McDonald MW, Dey A, Jiang M, Lacefield JC (2016). High Intensity Aerobic Exercise Training Improves Deficits of Cardiovascular Autonomic Function in a Rat Model of Type 1 Diabetes Mellitus with Moderate Hyperglycemia. J Diabetes Res.

[9] Sanches IC, Conti FF, Bernardes N, Brito J de O, Galdini EG, Cavaglieri CR (2015). Impact of combined exercise training on cardiovascular autonomic control and mortality in diabetic ovariectomized rats. J Appl Physiol.

[10] Nakos I, Kadoglou NPE, Gkeka P, Tzallas AT, Giannakeas N, Tsalikakis DG (2018). Exercise Training Attenuates the Development of Cardiac Autonomic Dysfunction in Diabetic Rats. In Vivo.

[11] Souza ED, Peixoto JVC, Rank C, Petterle RR, Fogaça RTH, Wolska BM (2023). Effects of High-Intensity Interval Training and Continuous Training on Exercise Capacity, Heart Rate Variability and Isolated Hearts in Diabetic Rats. Arq Bras Cardiol.

