# Supplementary material for: Diabetes Mellitus, Exercício Físico e Variabilidade da Frequência Cardíaca
Source: Arq Bras Cardiol. 2023 Jan 31;120(1):e20220902. [Article in Portuguese] doi: 10.36660/abc.20220902 (PMC10389116; doi:10.36660/abc.20220902)
Supplement: Translation

# Translation: Diabetes Mellitus, Physical Exercise and Heart Rate Variability

**Authors:** Gustavo Augusto Ferreira Mota, Mariana Gatto, Luana Urbano Pagan, Suzana Erico Tanni, Marina Politi Okoshi

**Affiliations:** 1 Brazil Departamento de Clínica Médica – Faculdade de Medicina de Botucatu – Universidade Estadual Paulista (UNESP), Botucatu, SP – Brazil

**Keywords:** Diabetes Mellitus/prevention and control, Exercise, Heart Rate, Risk Factors, Obesity, Sedentary Behavior, Aged

The prevalence of diabetes mellitus (DM) has grown in recent decades.^1^Risk factors for developing DM have been well established in clinical studies. Among these, sedentary lifestyles, aging and obesity stand out.^2^DM, defined by increased plasma glucose concentration, is commonly accompanied by cardiovascular alterations such as hypertension, coronary disease, heart failure, and cardiac autonomic neuropathy.^3^As cardiovascular diseases are currently the main causes of death and morbidity, studies aimed at better understanding the pathophysiological mechanisms inherent in DM are of great importance.

Cardiac autonomic neuropathy is characterized by changes in fibers that innervate the heart and blood vessels.^4 , 5^In this condition, changes in nerve function lead to reduced heart rate variability and altered baroreflex sensitivity.^6^Physical exercise is considered an important non-pharmacological therapy to help prevent and control DM and its clinical consequences.^7^Researchers have recently observed that exercise can also prevent and treat cardiac autonomic neuropathy.^7^

Physical exercise increases heart rate variability and improves autonomic function under physiological and pathological conditions.^8^Experimental studies in DM have shown that exercise decreases bradycardia and improves parasympathetic nervous system function.^9 , 10^However, the effects of exercise on heart rate variability in DM have not been fully clarified. Souza Neto et al.^11^investigated, in diabetic rats, the effects of physical exercise on heart rate variability, functional capacity, and cardiac functional variables obtained in preparations of isolated perfused hearts. The authors used the experimental model of type 1 DM induced by streptozotocin. The animals were subjected to two types of exercise: high-intensity interval training (HIIT), alternating cycles of 1:1 min between 50% and 90% of maximum exercise capacity, and continuous training at 70% of maximum exercise capacity. Rats without DM and rats with DM without training constituted the control groups. Electrocardiograms were recorded for one hour without the restriction of the animals. After 4 weeks, HIIT induced greater improvement in functional capacity than continuous training in control and diabetic rats. Cardiac function was reduced in DM groups and was not influenced by physical exercise. DM groups had lower heart rates than controls, regardless of performance and type of exercise. Despite the lower heart rate in diabetic rats, heart rate variability did not differ between DM and control groups.

As the authors emphasized, the short duration of the study^11^may have been a limitation of the study since effects resulting from DM and physical exercise on heart rate variability could have occurred if there had been a longer observation time.

Controversial results with respect to the literature show the need for additional studies to clarify the effects of high-intensity physical training and continuous training on heart rate variability during DM.
